# Glass Fibre-Reinforced Composite Post and Core Used in Decayed Primary Anterior Teeth: A Case Report

**DOI:** 10.1155/2011/864254

**Published:** 2011-09-21

**Authors:** Leena Verma, Sidhi Passi

**Affiliations:** Department of Pedodontics, Dr HSJ Institue of Dental Sciences and Research, 134109 Chandigarh, India

## Abstract

Aesthetic requirement of severely mutilated primary anterior teeth in the case of early childhood caries has been a challenge to pediatric dentist. Among restorative treatment options, prefabricated crown and biological and resin composite restoration either by means of direct or indirect technique are mentioned in the literature. This paper presents the clinical sequence of rehabilitation of maxillary anterior primary teeth. Endodontic treatment was followed by the placement of a glass fibre-reinforced composite resin post. The crown reconstruction was done with composite restoration. Resin glass fibre post has best properties in elasticity, translucency, adaptability, tenaciousness, and resistance to traction and to impact. Along with ease of application, fiber can be used as an alternative to traditionally used materials in the management of early childhood caries.

## 1. Introduction

The healthy oral cavity is a primary requisite for beautiful looks. Despite the fact that it is largely preventable, dental caries is the most common chronic disease of childhood [1]. Caries in very young children known as early childhood caries may be defined according to the American Academy of Pediatric Dentistry “as the presence of one or more decayed, missing (due to caries), or filled tooth surfaces in any primary tooth in a child 71 months of age or younger” [2]. Clinical examination of this condition discloses a distinctive pattern, and the teeth most often involved are the maxillary central incisors, lateral incisors, and the maxillary and mandibular 1st primary molars. The maxillary primary incisors are the most severely affected with deep carious lesions usually involving the pulp. In extreme cases, early childhood caries can even lead to total loss of the crown structure. Until very recently, the only treatment option for early childhood caries has been extraction of the affected primary anterior tooth, which resulted in severe coronal destruction. 

 The early loss of primary anterior teeth may result in reduced masticatory efficiency, loss of vertical dimension, development of parafunctional habits (tongue thrusting, speech problems), esthetic-functional problems such as malocclusion and space loss, and psychologic problems that can interfere in the personality and behavioral development of the child [3].

Aesthetic requirement of severely mutilated primary anterior teeth in the case of early childhood caries has been challenge to pediatric dentist. Among restorative treatment options, prefabricated crown and biological and resin composite restoration either by means of direct or indirect technique are mentioned in the literature. A post and core is a dental restoration used to sufficiently build up tooth structure for future restoration with a crown when there is not enough tooth structure to properly retain the crown, due to loss of tooth structure to either decay or fracture. An anchor placed in the tooth root following a root canal to strengthen the tooth and help hold a crown in place. 

 In cases where teeth are severely decayed, endodontic treatment and placement of intracanal posts or retainers become necessary before crown restoration. Posts may be constructed of a variety of materials, including resin composite, metal, and biologic material [4]. 

In recent years, various types of fiber reinforcement have come into widespread use as an alternative to cast or prefabricated metal posts in the restoration of endodontically treated teeth [5]. The advantages of using reinforced fiber to construct an intracanal post include resin composite crown reinforcement, translucency, and relative ease of manipulation [5, 6]. 

 This paper presents a case report of 4-year-old male with severely decayed maxillary anterior teeth that were restored with glass fibre-reinforced composite resin posts (GFRC), a new generation of fiber posts composed of densely packed silanated E glass fibers in a light curing gel matrix. The fibers are 7 to 10 micrometers in diameter and are available in a number of different configurations, including braided, woven, and longitudinal. The lower flexural modulus of fiber-reinforced posts (between 1 and 4 × 106 psi) measures closer to that of dentin (2 × 106 psi) and can decrease the incidence of root fracture. 

## 2. Case Report

**Figure 1:**
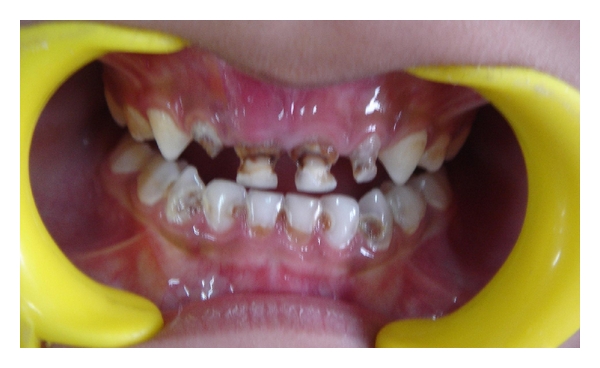
Preoperative intraoral photograph of the patient.

**Figure 2:**
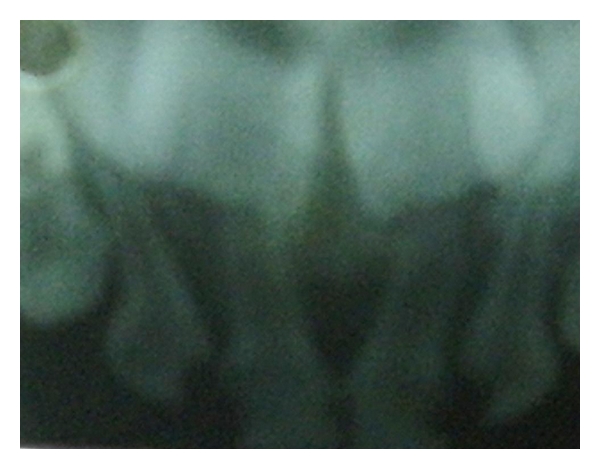
Intraoral periapical radiographs revealed pulp involvement of 52, 51, 61, and 62.

**Figure 3:**
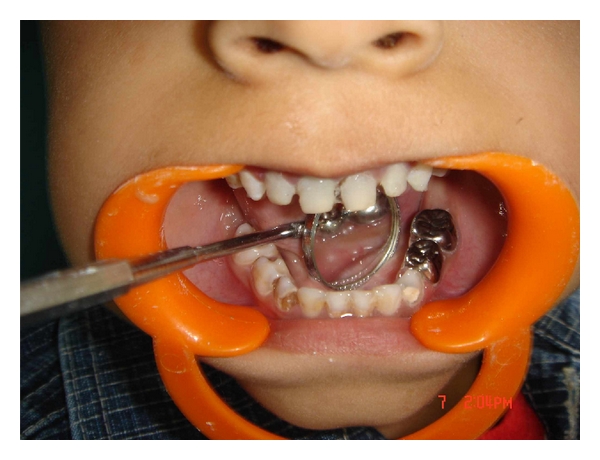
74 and 75 were grossly carious and were indicated for pulpectomy followed by a stainless steel crown.

A 4-year-old male patient reported to the Department of Pedodontics and Preventive Dentistry with a chief complaint of decayed upper front teeth. Patient's medical history was noncontributory. Patient's mother gave a history of breast feeding for 1 year after which the child was bottle fed for 3 years. Intraoral examination revealed a complete set of deciduous dentition. It was observed that 55, 52, 51, 61, 62, 65, 71, 72, 73, 74, 75, 81, 82, 83, 84, and 85 were affected by dental caries (Figure 1). Intraoral periapical radiographs revealed pulp involvement with 52, 51, 61, and 62 (Figure 2). Diet analysis, counseling, and oral prophylaxis were done. 74 and 75 were grossly carious and were indicated for pulpectomy followed by a stainless steel crown (Figure 3). 52, 51, 61, and 62 were indicated for pulpectomy, followed by glass fibre-reinforced composite resin posts and composite buildups. 55 and 65 presented with pit and fissure caries and were indicated for composite resin restorations. 

The treatment plan was divided into 2 steps for 51, 52, 61, and 62.

**Step 1**

Endodontic phase.

**Step 2**

Construction of the restoration.

### 2.1. Step  1: The Endodontic Phase

**Figure 4:**
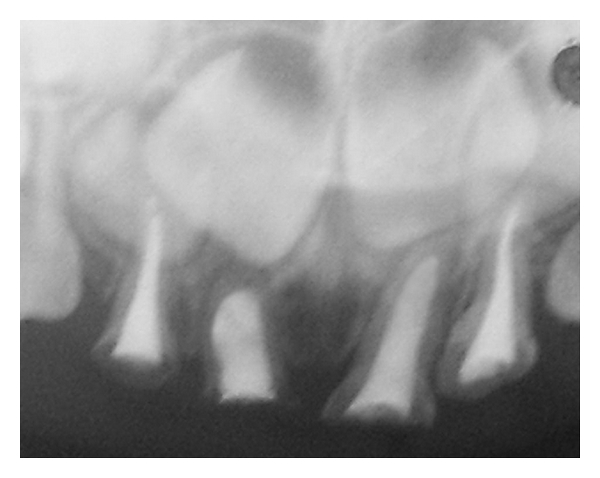
IOPA showing complete obturation of 52, 51, 61, and 62.

An infraorbital block was administered for 61 and 62, and labial and palatal infiltration was carried out for 51 and 52. Gross carious lesions were removed with a no. 330 round carbide steel bur. Unsupported enamel was not removed so as to preserve as possible. The pulp chamber was opened and working length determination IOPA was taken with a no. 10 K-file.The pulp tissue was extirpated using no. 10–no. 35 K-files. After irrigation with copious amounts of 2.5% NaOCl and normal saline, the root canal was dried using paper points. A thick mix of Endofloss paste was then condensed with lentulo spiral into the canal. The obturated material was allowed to set for 10 minutes (Figure 4).

### 2.2. Step  2: The Construction of Restoration

The post space was prepared 1 week after the endodontic treatment was completed. The post space was created by removing approximately 4 mm of Endofloss material using a thin straight fissure bur. All visible Endofloss cement on the walls of the post space was removed. 

**Figure 5:**
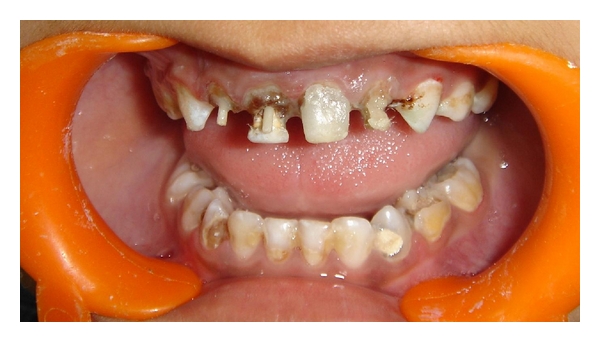
Glass fibre-reinforced composite post (GFRC) inserted.

The prepared post space was then cleaned with saline, air dried, and acid etched with 37% phosphoric acid for 15 seconds. This space was rinsed and air dried with oil-free compressed air. A light-cured bonding agent was brushed on the etched surface and uniformly dispersed by a compressed air blast. It was then light cured with for 20 seconds. The GFRC post was then cured for 20 seconds in order to gain rigidity, before insertion into the post space. Light cured flowable composite resin was then inserted into the canal chamber after which the GFRC post was inserted (Figure 5). The fiber post and composite were then cured with together for 60 seconds. The coronal portion of the glass fibre-reinforced composite post was splayed to increase the surface area for the retention of the core. 

**Figure 6:**
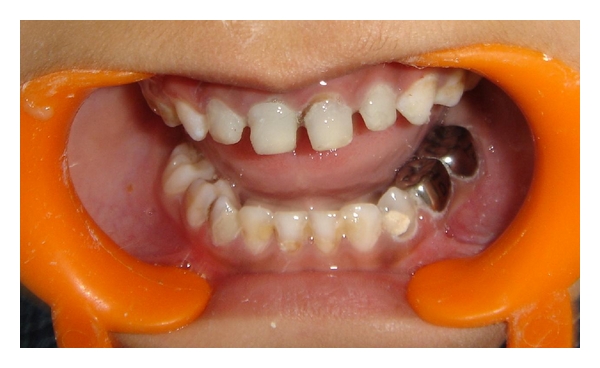
Postoperative intraoral photograph of the patient.

The coronal enamel was then etched for 20 seconds, rinsed with water and air dried followed by application of bonding agent—which was then light cured. The coronal post was then covered with the flowable composite for core build up, followed by light curing it for 60 seconds, and finally teeth were restored with hybrid composite. The final finishing and polishing was done with finishing burs. Occlusal interferences in normal and paranormal mandibular movements were removed (Figure 6).

## 3. Discussion

Esthetic restoration of primary teeth has long been a special challenge to pediatric dentists. When there is severe loss of coronal tooth structure, the use of posts placed inside the canal after endodontic treatment will give retention, provide stability to the reconstructed crown, and withstand masticatory forces in function. There are a variety of root posts used in pediatric dentistry. A resin composite post building up directly, resin composite short post placement, alpha, or omega shaped orthodontic wires, stainless steel pre fabricated posts, nickel- chromium cast posts with macroretentive elements, natural teeth from a tooth bank or reinforced fibers. Prefabricated posts are fast, cheap, and easy to use, but they do not take into account the individual shape of the root canal. Although metal posts are indicated for primary teeth but because of their color metal post do not meet the esthetic requirement. Moreover these may cause problems during the course of natural exfoliation. Composite post provides satisfactory esthetics; however, there is risk of loss of retention owing to polymerization shrinkage [6]. The use of omega-shaped stainless orthodontic wire as an intracanal post is also simple. However, the wire is unable to adequately adapt to the canal form, because it is not the exact copy of the canal.

The development of the fibre-reinforced composite technology has brought a new material into the realm of metal-free adhesive esthetic dentistry. Different fiber types such as glass fibers, carbon fibers, Kevlar fibers, vectran fibers, and polyethylene fibers have been added to composite materials. Carbon fibers prevent fatigue fracture and strengthen composite materials, but they have a dark colour, which is undesirable esthetically. Kevlar fibers made of an aromatic polyamide increase the impact strength of composites but are unaesthetic, and, hence, their use is limited. Vectran fibers are synthetic fibers made of aromatic polyesters. They show a good resistance to abrasion and impact strength, but they are expensive and not easily wielded [7]. Polyethylene fibers are esthetic but their flexural strength is less as compared to glass fiber-reinforced composite posts. The biological posts require the availability of a tooth bank and are still subject to new studies for future conclusions [8]. 

 Glass fiber-reinforced composite resin posts (GFRC) are new to the pediatric world and can be used as an alternative to the other post systems. The properties of fiber- reinforced posts are dependent on the nature of the matrix, fibers, interface strength, and geometry of reinforcement. The advantages of this material over the older fibers are greater flexural strength (1280 MPa), and over 650 MPa of the older fibers, greater ease of handling, can be used in high stress-bearing areas and can be bonded to any type of composites. Scanning electron microscopic (SEM) evaluation has shown clearly the formation of a hybrid layer, resin tags, and an adhesive lateral branch. Successful bonding minimizes the wedging effect of the post within the root canal, requires less dentin removal to accommodate a shorter and thinner post, and leads to lower susceptibility to tooth fracture. These posts are placed in cervical one third of the canals, to avoid interference with the process of permanent tooth eruption [9]. When compared to other fibres, they are almost invisible in resinous matrix. Due to these reasons, they are the most appropriate and the best esthetic strengtheners of composite materials [10].

## 4. Conclusion

The number of endodontic procedures has increased steadily in the past decade with highly predictable results. Therefore, restoration of teeth after endodontic treatment is becoming an integral part of the restorative practice in dentistry. The treatment described in the case report is simple and effective and represents a promising alternative for rehabilitation of grossly destructed or fractured primary anterior teeth. This technique of glass fibre-reinforced composite resin post and core has shown promising results and has presented the pediatric dental world with an additional treatment option.
